# Exploring the situational motivation of medical specialists: a qualitative study

**DOI:** 10.5116/ijme.5a83.6025

**Published:** 2018-02-26

**Authors:** Stéphanie M.E. van der Burgt, Rashmi A. Kusurkar, Gerda Croiset, Saskia M. Peerdeman

**Affiliations:** 1 VUmc School of Medical Sciences, Research in Education, Amsterdam, the Netherlands

**Keywords:** Exploring, situational motivation, medical specialists, qualitative study, The Netherlands

## Abstract

**Objectives:**

The aim was to obtain insight into the factors in the work environment that motivate or demotivate a medical specialist during his/her working day.

**Methods:**

A qualitative ethnographic design was used, and a constructivist approach was adopted with the Self-Determination theory of motivation as a framework. Six medical specialists from VU University Medical Center in the Netherlands, recruited through convenience, snowball, and purposive sampling, were shadowed for one day each. Data were transcribed and open-coded. Themes were finalized through discussion and consensus.

**Results:**

Sixty hours of observation data identified motivating and demotivating factors categorized into four themes that are important for specialists’ motivation. Informational technology issues are demotivating factors. Working with colleagues can be both a motivating and demotivating factor, e.g., filling in for each other through feelings of relatedness was motivating. Being in control of one’s planning through feelings of autonomy was motivating. Furthermore, patient care and teaching, especially in combination, stimulated specialists’ motivation. Regarding the design of the study, we found that situational motivation is indeed observable.

**Conclusions:**

The basic psychological needs autonomy, competence, and relatedness are important for specialists’ motivation. Investing in a more motivating, open, transparent, and basic-needs- supportive work environment for medical specialists is necessary. Keywords: Continuing professional development, motivation, medical specialists, self-determination theory, qualitative research.

## Introduction

The medical system is becoming more fragmented and more efficiency-minded. This continuously changing work environment, changing societal demands, changing levels of expertise, and social and personal changes demand the continuous adaptation of medical specialists during their workday.^1^^-^^3^ Changes that occur faster than people can adjust or develop to, lead to more adverse events and less patient safety. This profoundly impacts society’s trust in the healthcare system.^4^ In the Netherlands, the most recent study reports 970 preventable adverse events in hospitals per year.^4^^,^^5^ This necessitates medical specialists to face the challenge of learning throughout their career, maintaining their professional competence and keeping track of and responding to changes in their professional content.^4^^,^^6^

Motivation has been found to play an important role in the learning and performance of health professions students.^6^ We expect that it also plays an important role in the learning and performance of medical specialists, particularly because motivation for work also appears to be positively associated with the hours that health professionals invest in continuing education.^6^^-^^10^ Motivating and demotivating factors for work motivation have been found at the individual, departmental, institutional, and societal levels.^4^^,^^6^^,^^11^ 

While research has provided insight into the social and intrapersonal antecedents of motivation, the relationship between different hierarchical levels of motivation has not been sufficiently investigated.^12^ Little is known about the dynamic interplay between the contextual and situational motivation of medical specialists.^12^^,^^13^ Medical specialists’ motivation has also not been studied previously. Our study aims to investigate the interplay of situational and contextual motivation and how factors that trigger feelings of autonomy, competence, and relatedness support specialists’ situational motivation. Knowing these factors can provide the opportunity to create the best possible environment for specialists to work in, to support their situational motivation directly and their contextual motivation indirectly. When a specialist is motivated at the contextual level, he/she is more likely to have long-term motivation for medical practice.^6^^,^^13^^,^^14^ This is expected to benefit the delivered healthcare. This leads us to the following research question:

§  Which factors in the work environment motivate or demotivate a medical specialist during his/her working day?

### Theoretical framework

Self-Determination Theory (SDT) classifies different types of motivation, focusing on the quality, along with a dynamic continuum.^15^ Controlled motivation (CM) makes a person pursue an activity to obtain a certain reward or avoid a certain loss or punishment; autonomous motivation (AM) exists when a person pursues an activity out of personal interest.^12^^,^^15^^,^^16^ There is evidence from medical education that the best quality motivation, AM, is associated with better learning, better academic performance, and most importantly, better patient care.^17^^-^^20^ Within SDT, three basic psychological needs have been distinguished: autonomy (experiencing a sense of volition), perceived competence (experiencing improvement of skills), and relatedness (feeling connected with peers and role models).^12^^,^^15^^,^^16^ The fulfillment of these three needs is necessary for the optimal development of AM.

In addition to the description of the continuum, SDT endorses a hierarchical model of motivation with three levels: global, contextual, and situational.^12^ At the global level, the individual is seen as having developed a global (or general) motivational orientation to interact with the environment in an intrinsic, extrinsic, or amotivation way.^12^^,^^15^^,^^16^  Contextual motivation concerns the motivational orientations that individuals develop toward each life context (like education, work, leisure and interpersonal relationships).^12^^,^^15^^,^^16^ In this study, contextual motivation is the motivation that a medical specialist has for his/her job, so for medical practice in general. In our setting, a medical specialist is a physician with a completed specialty training. Situational motivation refers to the motivation individuals experience at a particular moment or in a particular situation -the “here-and-now” of motivation- and is likely to be influenced by social factors.^12^^,^^15^^,^^16^ In this study, situational motivation refers to the motivation for the different tasks that a medical specialist must handle during a day, e.g., handling patients, doing the administrative work, and attending meetings. The three levels of motivation can have reciprocal effects on each other.^12^ This is because repeatedly engaging in autonomously motivating activities (at the situational level), together with experiencing their beneficial consequences, plays a role in facilitating contextual AM.

To engage physicians in staying motivated, an appreciative inquiry into factors important for their motivation at work on a day to day basis (situational motivation) is necessary.^21^^,^^22^ We, therefore, decided on a research plan for an observational study.

Within the field of motivation research, there is a call to use qualitative methods instead of the current over-reliance on self-reported questionnaires.^23^ In the absence of tried and tested methods for collecting data for qualitative research in motivation, we decided to conduct this qualitative study as an initial study.

## Methods

### Study design

To identify as many factors as possible, a qualitative design was used, and an ethnographic approach with observations was adopted within the constructivist paradigm.^21^^,^^22^ In this approach, there is acceptance of reality and meaning as relative, produced through the interaction between the researcher and the researched, acknowledging the subjectivity of the researchers producing accounts of a social phenomenon.^21^^,^^22^ In this study, SB observed the specialists in their context through her training as a sociologist and blended this perspective with those of study participants and an “insider informant” engaged in the collaborative analysis process.^21^^,^^22^ The insider informant was the author SP; she is a medical specialist.

### Sample

Through convenience, snowball, and purposive sampling six medical specialists were selected. This included different disciplines to provide for the transferability of findings and identification of common factors across disciplines. Snowball sampling was done by asking the participants to suggest their peer specialists for participation. Snowball sampling is a non-probability sampling technique, often used in sociology, which is appropriate to use in research when the members of a population are difficult to locate or, as in this case, “hard to find” specialists willing to participate.^24^ Snowball sampling can also be used for exploratory purposes.^24^ We sampled until sufficiency was reached, i.e., sufficiency for gathering the appropriate information to answer the research question.^25^^,^^26^ After four observations, we found that situational motivation can be studied through observations. For extra security, two more observations were conducted. For this initial study six participants were sufficient because it is the first attempt at observational qualitative research.  The medical specialists who participated in this study included a neurologist, an ENT-surgeon, a radiotherapist, a psychiatrist, a geriatrician, and a general surgeon. Five specialists were males, one was female, and the average age was 49 years.

Ethical approval for this study was obtained from the local Institutional Review Board of the VU University Medical Center, Amsterdam, the Netherlands. Informed consent was gathered from all participants prior to conducting the observations, acknowledging the anonymized use of their statements in this study. However, informed consent was with minimal disclosure (offering generic rather than specific study information to help minimize the observer effect in field research) to prevent participants from altering specific behaviors.^22^

### Setting

This study took place in VU University Medical Center, Amsterdam, the Netherlands. Every participating specialist had the opportunity to choose a day that suited him/her. SB observed each participant for one day. This also led to a variety of types of days; one participant on a management day, one on an education day, one on a day at the clinic, one on a supervision day, and two on a day that was scheduled with different tasks. All specialists stated that the observation day was representative of a typical workday.

### Data collection

All medical specialists were shadowed by SB for one day each. Given that most human behavior occurs within a context that may be informed by previous contexts and their activities, one researcher was responsible for collecting data from each participant.^21^^,^^22^ This is to ensure that the previous contexts could be considered in the interpretation of the later ones.^21^^,^^22^ The observation started at the moment the medical specialist entered the hospital, and ended when the specialist left the hospital at the end of the day. This resulted in approximately 10 hours of observation per medical specialist. The focus of the observations was to unravel motivating and demotivating factors during a workday. Therefore, SB observed what happened to the mood of a medical specialist -whether an event, activity, or situation was motivating or demotivating. More motivating was defined by; when a specialist seemed cheerful, happy and relaxed by observing laughter, smiles, relaxed appearances, active attitude/ posture, or hearing a specialist say that something is nice, positive, motivating, or satisfactory. More demotivating was defined by; when a specialist seemed grumpy, irritated, tired, unhappy, stressed by observing frowns, shaking their head, or hearing a specialist curse, sigh, or say that something is negative, irritating, frustrating, or demotivating. Brief contemporary notes were taken during the observations, and extensive field notes were written up immediately after each daily observation to create a thick description.^25^ Besides field notes, the researcher kept a reflective diary to ensure a certain distance from the observation notes and to ensure the validity of the collected data.^25^ At the end of the observation day, participants were asked about their thoughts on observed situations for stimulated recall and to ensure the trustworthiness of the gathered data. This strengthened the internal validity of the data because the observation could be discussed and viewed through the perspective of the participant.

### Data analysis

All qualitative data were transcribed and coded in Atlas.ti. The transcripts were open-coded in a constant comparative manner by attaching keywords to all relevant text fragments. SB familiarized herself with the data and coded all observational notes. SB did this after every observation, so she knew what to focus on for the next observation. The first and fourth observation were also coded independently by RAK Whenever there were differences in coding, these were discussed until a consensus was reached. We finalized themes through selective coding, iterative discussion, and consensus in the full research team, which also ensured the objectivity of the data analysis.

### Reflexivity

Out of four researchers in this study, one is a sociologist, and three are medical doctors experienced in research in education and motivation, of which one is a clinical specialist. This research team set up was important in designing the qualitative data collection technique and questions, and thinking proactively about all the ethical aspects that might be involved while making the observations. We tried to balance our research findings through the different analytical perspectives (a sociologist’s perspective, two doctors’ perspectives, and a practicing clinical specialist’s perspective). Having three physicians in the team helped to understand the perspective of the community of physicians, ensuring that important findings were not missed by the sociologist in the coding of the data. Having a clinical specialist on board helped to understand the findings from the perspective of the people being observed as well as to put it in the right context. The sociologist had no familiarity with the clinical discourse, and this helped to move beyond the ideologically driven account of the informants’ doings. This helped us to optimize the analysis of the data better. Also, the observer being a sociologist made for absence of inherent power dynamics in the relationship of the observer toward the participants. In the spirit of reflexivity, we acknowledge our assumption that motivation can change. However, this assumption is theoretically supported.^12^^-^^14^^,^^19^^,^^27^^,^^28^

## Results

Through the analysis of the data, factors were identified and could be classified into four themes to be of importance for medical specialists’ motivation for their work. These will be described below, supplemented with quotes from medical specialists or descriptions of situations that show motivation or demotivation.

### Interaction with colleagues

Interaction with colleagues can be both motivating or demotivating. The specialists experienced feelings of relatedness (or connectedness) that supported their motivation when colleagues were willing to fill in for each other and could consult with or just talk to colleagues. Three out of the six specialists told the observer that “it is really important and nice to be able to talk to colleagues about work or sometimes private things.” This was also seen in every medical specialist through laughter and during private talks and making jokes with colleagues. The observer perceived that the participants' sense of relatedness and connectedness were strengthened as observed by their engagement in private talks and joke-telling with colleagues.

Then the specialist gets summoned by his “boss”, who tells him that he is going to take over the specialists’ morning round. The specialist laughs and tells the observer:

“This is a present from the boss”. (specialist 4, male)

This quote shows that there is more joy in work when colleagues appreciate each other and are willing to fill in for each other when necessary. This specialist was particularly happy because the head of the department cleared an activity from his schedule, as he saw that it was an impossible one to accomplish that day.

Meanwhile, a colleague walks in and asks how it is going today, also to talk things through about work and to just have a chat. Jokes are made, and there is laughter. (specialist 5, female)

The quote above shows relatedness between colleagues and a nice or fun way of working together and being able to discuss work or private matters.

However, when a colleague did not communicate properly, it decreased feelings of relatedness and was demotivating. Medical specialists primarily managed their frustration by sharing it with their colleagues, often the frustration about one event that occurred several times during a day.

A specialist hears that a close colleague is not in today. Frustrations are expressed by making a face and saying: “Really? He is not in today? Gosh, this keeps happening, and I am left here clueless; he always does this. He leaves everyone, doing his work”. (specialist 5, female)

This quote shows that one of the specialists colleagues did not keep her in the loop of his whereabouts, and this frustrates her because 1) it keeps happening and 2) it gets her and other colleagues into trouble regarding his and their work. It also provides an imbalance in the working relationship between these colleagues, which will decrease their feelings of relatedness.

The specialist tells me that he was quite angry the other day, about the way that his patient was treated by some colleagues. These were colleagues from another specialty.(specialist 3, male)

His patient being treated, in his opinion, badly by colleagues from another specialty creates friction between the specialists. This friction decreases their feelings of relatedness. In addition, when specialists do not work together properly it can decrease feelings of competence. This specialist was not able to provide his patient with the (quality of) care he wanted.

### Autonomy in organizing one’s own time

Being in control of one’s planning through feelings of autonomy was motivating. The quote below states that this specialist consciously chooses how he divides his time and days. Especially when he has the task of supervising residents or medical students, as he feels the need to be flexible on these days.

“I try to organize my schedule in a way so that I can be present when needed for my patients or students. This means minimizing fixed appointments”. (specialist 4, male)

This is an illustration of a practical way of organizing your own time to match your preferences.

It also appeared that organizing things properly at home ensured that specialists were able to focus on their work better. Several specialists told the observer that they have more piece of mind when they know that their children are taken care of during the day. However, when medical specialists are not able to organize their own time or day, it is demotivating. They experience a loss of autonomy. This was told to the observer and seen in every specialist continuously throughout the day, mostly when it involved patient care. Specialists feel like they do not have sufficient time for their patients. Administrative work, meetings, and inefficient planning and communication structure take too much time away from patient care.

### Informational technology issues

Issues with informational technology (IT) were demotivating. Initially these issues might be irritating, but if they continue to exist, they become a demotivating factor. It is demotivating because when something does not work properly, specialists do not feel like they can work with all IT systems or items they need. This decreases their perceived competence.

“Damn it stupid computer system”. This is followed by a sigh and grumble by the specialist. A smile appears when it looks like the system is working again. (specialist 2, male)

This medical specialist gets frustrated when his computer system suddenly does not work anymore. He became grumpy and almost angry, which is demotivating in his work.

These issues were seen every observation day, and all specialists grumbled or complained about it. The specialists said that in the moment when something does not work, it is irritating, but you deal with it. However, if they have to deal with it every day, or very frequently, it keeps them from doing their job properly and how they would like to.

### Patient care and teaching

Patient care in itself motivates medical specialists. During the care of their patients, every observed medical specialist appeared more energetic, cheerful, and there was much laughter. All specialists were willing to put in some extra effort when it came to taking care of a patient or teaching during patient care. Transferring knowledge to residents seemed to be a motivating factor as well. This theme emerged through the observations; only a few specialists mentioned it explicitly. One specialist stated:

“The thing that motivates me the most is doing the follow up together with the residents. So that is a combination of patient care and teaching”. (specialist 1, male)

This is an explicit statement on the motivating effect of the factors patient care and teaching. And in this example, it is even the combination of the two that is the most motivating: probably because within this combination all three basic psychological needs come together. The need for autonomy is fulfilled because this specialist is taking care of his patient and teaching his student in the way that he feels is best. The need for competence is fulfilled because of knowledge transfer and being in the lead, and the need for relatedness is fulfilled because the specialist can relate to his patient and his resident.

## Discussion

The preliminary results of this study indicate that factors that stimulate autonomy and relatedness motivate medical specialists. Demotivating factors found were difficult collaboration with colleagues and technical issues. These factors thwart feelings of relatedness and perceived competence. Also, thwarting feelings of autonomy, for example not being able to organize one’s time schedule, is demotivating. Tasks that were the most motivating were patient care and teaching. Teaching could be implicit or explicit. However, most specialists mentioned implicit teaching or just knowledge transfer. Hence, this study points to the relevance of the fulfilment of autonomy, competence, and relatedness in the daily practice of medical specialists. This has been demonstrated previously in other settings and professions, like teachers,^29^ nurses,^9^ and pharmacists.^10^^,^^27^^,^^28^ When these basic psychological needs are thwarted, AM is unlikely to be reached. This means that when we want to assist medical specialists in staying motivated for medical practice, there needs to be an environment that stimulates their basic needs. This study shows that at this moment it is not possible to fulfil their basic psychological needs.

The results show that medical specialists feel the need for more autonomy during their workday. According to the SDT, professionals who are autonomous or are supported to be autonomous have a higher level and better quality of motivation.^30^ This can lead to a better quality of work performance, which is better for the delivered health care. This provides arguments for the implication that medical specialists are professionals and should be as autonomous as possible within the context and culture of the organization of health care and in the hospital in which they work.

The next implication is the creation of a culture at the workplace where specialists can openly discuss their frustrations and address others’ behaviors and attitudes when necessary. Particularly because previous research suggests that causes contributing to the onset and continuation of poor performance also include organizational and cultural aspects.^4^^,^^21^ Furthermore, the larger healthcare system as a whole, as well as aspects related to learning and performance are causes that contribute to the onset and continuation of poor performance.^4^^,^^21^ The present results show that it is motivating to work in an environment where specialists feel related to each other and where there is adequate communication.

When specialists experience no IT issues, it is not motivating; it is just expected and considered normal to have no technical difficulties. Furthermore, every medical specialist in this study had at least some comment on the IT systems used in their work environment, whether they experienced difficulties or not. They were not content with the type of system they had to use. This thwarts their autonomy because they cannot decide on the system with which they work. This is chosen for them by someone else in the medical center. Following this, a third implication of this research is to invest in training specialists to work with all technical equipment. Next to this, let specialists, as the end users, have a say in the decisions about the IT systems. Also have IT experts on standby to support them when there are technical difficulties. Specialists do not feel competent when their technical equipment is unknown to them or when it does not work properly, which is demotivating. Furthermore, learning and development are two sources of energy for professionals and also for medical specialists, which can stimulate motivation.^7^^,^^31^ Knowing which factors motivate a medical specialist provides the opportunity to create the best possible environment for specialists to work in. In the longer term, this can enhance their contextual motivation (overall motivation for their work), which leads to a higher level of professionalism.^9^^,^^21^ This is because, as mentioned previously, repeatedly engaging in autonomously motivating activities (at the situational level), together with experiencing their beneficial consequences will play a role in facilitating contextual autonomous motivation.

### Study limitations

 The main limitation of this study is that using snowball sampling as the sampling technique could have led to a more motivated sample of medical specialists than the general population. More motivated specialists or specialists interested in the subject of motivation and professional development could have been more drawn to participate, or the participants may have identified people who feel the same way they do, therefore not providing us with an adequate and representative sample. This could give a more positive image of the motivation of medical specialists. However, if we indeed have more motivated specialists in our sample, and still found these demotivating factors and barriers for medical specialists to do their work at an optimal level, we could say that these factors are very important. Maybe even more so for less motivated specialists, and that these factors may demotivate them even more. Although we acknowledge the potential limitation in our sample due to the use of snowball sampling, it is very difficult to use any other sampling method for this type of a study, as the participation in the study is voluntary. Snowball sampling works very well because participants learn about the study through a recommendation from a trusted colleague who has already participated in the study. Another limitation is that we did not triangulate the data with the participants, in the sense that we did not interview them. Interviewing participants is considered a way of ensuring the trustworthiness of the research study. As noted previously, however, in this study the participants were asked about observed situations for stimulated recall. This assured the trustworthiness of the data.

## Conclusions

Four main factors are found to be of importance for the motivation of medical specialists. The first is the interaction with colleagues, which provides relatedness. The second is autonomy in organizing one’s own time, which provides the feeling of being autonomous. The third factor is informational technology issues that thwart the need of feeling competent, and the fourth is patient care in combination with teaching in which all three needs are fulfilled. Thus, the basic psychological needs autonomy, competence, and relatedness are important underlying influences on motivation for medical specialists. Therefore, we recommend investing in a more motivating and basic-needs-supportive work environment for medical specialists. This means an environment with an open and transparent culture, where specialists feel autonomy to schedule their workday, connected to their colleagues, and properly equipped and supported for IT difficulties.

After this first attempt to describe the situational motivation of medical specialists, additional research will be planned to unravel the various elements of motivation further. The results of this study opt for more insight into the mechanism behind the motivation of medical specialists and also see whether the context of a non-academic hospital provides other important factors for motivation. Furthermore, research on how specialists try to keep up with the continuously changing work environment and what kind of support or interventions they would like to have. In this way, continuing medical education for medical specialists can be built up from an autonomy-supportive perspective.

### Acknowledgements

The authors would like to thank all medical specialists of VU University Medical Center, Amsterdam, the Netherlands who participated in this study.

### Conflict of Interest

The authors declare that they have no conflict of interest.
